# Altered Response Hierarchy and Increased T-Cell Breadth upon HIV-1 Conserved Element DNA Vaccination in Macaques

**DOI:** 10.1371/journal.pone.0086254

**Published:** 2014-01-23

**Authors:** Viraj Kulkarni, Antonio Valentin, Margherita Rosati, Candido Alicea, Ashish K. Singh, Rashmi Jalah, Kate E. Broderick, Niranjan Y. Sardesai, Sylvie Le Gall, Beatriz Mothe, Christian Brander, Morgane Rolland, James I. Mullins, George N. Pavlakis, Barbara K. Felber

**Affiliations:** 1 Human Retrovirus Pathogenesis Section, Vaccine Branch, Center for Cancer Research, National Cancer Institute, Frederick, Maryland, United States of America; 2 Human Retrovirus Section, Vaccine Branch, Center for Cancer Research, National Cancer Institute, Frederick, Maryland, United States of America; 3 Inovio Pharmaceuticals, Inc., Blue Bell, Pennsylvania, United States of America; 4 Ragon Institute of MGH, MIT and Harvard, Boston, Massachusetts, United States of America; 5 IrsiCaixa AIDS Research Institute-HIVACAT, Autonomous University of Barcelona, Barcelona, Spain; 6 Institucio Catalana de Recerca i Estudis Avancats (ICREA), Barcelona, Spain; 7 Department of Microbiology, University of Washington, Seattle, Washington, United States of America; 8 Department of Medicine, University of Washington, Seattle, Washington, United States of America; 9 Department of Laboratory Medicine, University of Washington, Seattle, Washington, United States of America; Boston College, United States of America

## Abstract

HIV sequence diversity and potential decoy epitopes are hurdles in the development of an effective AIDS vaccine. A DNA vaccine candidate comprising of highly conserved p24^gag^ elements (CE) induced robust immunity in all 10 vaccinated macaques, whereas full-length *gag* DNA vaccination elicited responses to these conserved elements in only 5 of 11 animals, targeting fewer CE per animal. Importantly, boosting CE-primed macaques with DNA expressing full-length p55^gag^ increased both magnitude of CE responses and breadth of Gag immunity, demonstrating alteration of the hierarchy of epitope recognition in the presence of pre-existing CE-specific responses. Inclusion of a conserved element immunogen provides a novel and effective strategy to broaden responses against highly diverse pathogens by avoiding decoy epitopes, while focusing responses to critical viral elements for which few escape pathways exist.

## Introduction

An ideal HIV vaccine should provide protection against all HIV-1 variants. Yet, HIV-1 is so variable that it may escape immunity provided by the vaccine candidates evaluated to date, due to intrasubtype or even intrahost diversity. To address this problem, approaches to maximize immunological strength and breadth are being explored, including strategies that use consensus, center-of-tree or ancestral sequences, multiple strains or mosaic immunogens, immunogens consisting of known epitopes from the database, and chimeric molecules expressing a selection of the most conserved epitopes from different clades of HIV [1]–[17].

In addition to sequence diversity, the presence of potential immunodominant “decoy” epitopes provides another hurdle in the development of effective HIV vaccines. Accumulating evidence indicates that immunodominant epitopes may constitute an impediment for the production of effective universal HIV vaccines [18]–[31], as subdominant epitopes within HIV proteins have generally been associated with virologic control [19], [22]. The use of any complete HIV-1 protein comprises an immunogen containing both variable as well as conserved regions. Since variable sequences can mutate to escape immune responses while retaining function, and can focus the T cell responses away from the conserved epitopes, they should be excluded from the immunogen design [32]. This would target the immune responses to nearly invariable proteome segments, many of which are essential for the survival of the virus, and prevent responses against variable segments and potentially immunodominant “decoy” epitopes [32], [33]. The conserved elements used in our work were selected using different criteria compared to those used by others [11], [12], [16], [17], [34]–[36], as we have focused on both stringent conservation and association of particular elements with virologic control [32], [33].

**Figure 1:**
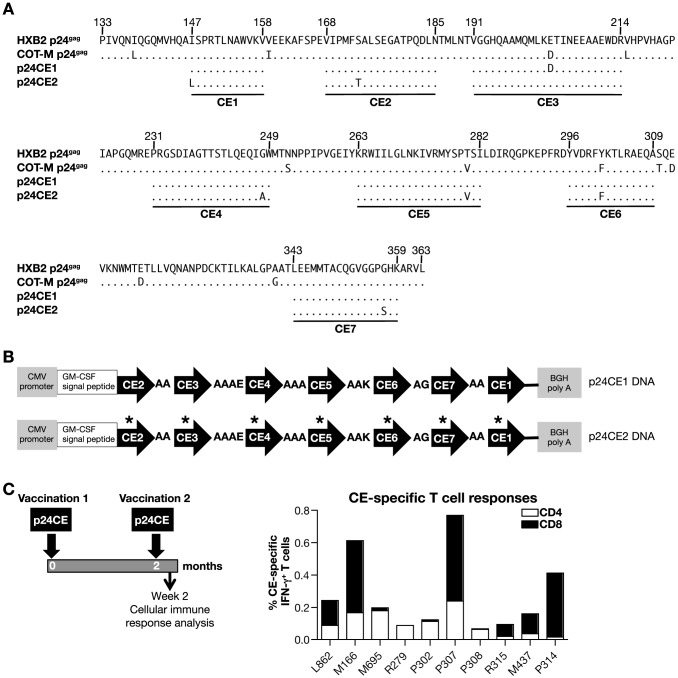
p24CE DNA vaccine is immunogenic in macaques. (**A**) Alignment of the amino acid (AA) sequence of the 7 CE represented in the p24CE1 and p24CE2 proteins with HXB2 and COT-M p24^gag^ proteins. The toggled AA in each CE is shown. The numbering of the AA in HXB2 p24^gag^ protein is according to the HIVDB (www.hiv.lanl.gov/). (**B**) The p24CE (p24CE1 and p24CE2) proteins are composed of 7 CE which were collinearly assembled in the order CE2-3-4-5-6-7-1 to avoid a strongly hydrophobic N-terminal CE1 were connected via short linker sequences designed for efficient proteolytic cleavage and contain the human GM-CSF signal peptide at the N-terminus as described [42]. Plasmids SP-p24CE1 and SP-p24CE2 contain the GM-CSF signal peptide at the N-terminus of p24CE to promote secretion of the p24CE proteins. The CE1 and p24CE2 proteins differ by 1 AA per CE (see **Figure 1A**), indicated by asterisks. (**C**) CE-specific cellular immune responses induced upon vaccination with p24CE. Macaques (N = 10) were vaccinated with p24CE DNA as outlined in the left panel and 2 weeks after the 2^nd^ vaccination the cellular immune responses were analyzed. Note, macaques M437 and P317 received prior 3 vaccinations with DNAs expressing the poorly immunogenic unmodified p24CE (see Table 1). The frequency of CE-specific IFN-γ^+^ T cells was measured using peptide pools composed of a mixture of 15-mer Group M peptides overlapping by 11 AA and 10-mer peptides overlapping by 9 AA covering both p24CE1 and p24CE2 proteins. The CE-specific CD4^+^ (open bars) and CD8^+^ (filled bars) T cells are shown.

The present work focused on Gag as a prototype vaccine, because Gag-specific T cell responses were found to correlate with control of viremia in clade B and C infected individuals [37]–[40]. Seven highly conserved elements (CE) were identified in HIV-1 p24^gag^ [[32], [33]; see **Figure 1**]. Indeed, a cross-sectional *ex vivo* study among HIV-1 infected individuals showed broad recognition of several CE in the context of wide HLA diversity and identified T cell responses of high functional avidity and broad variant reactivity [33], predominantly in controller individuals, suggesting an association between these T–cell responses and HIV control. We have engineered a prototype DNA vaccine expressing these 7 CE as a single protein (p24CE) and reported that human dendritic cells transfected with p24CE RNA were able to induce cellular responses in autologous T cells *ex vivo* at levels comparable to those induced by full-length Gag, but focused on CE [41]. We further reported that p24CE DNA vaccination induced strong cross-clade specific cellular and humoral immune responses in C57BL/6 mice [42]; these responses were of higher magnitude and recognized more CE than those induced by the complete Gag, despite their conservation within the intact Gag [42]. The poor responses to CE induced from the full-length p55^gag^ DNA vaccine suggested that this could be due to poor antigen processing or presentation, or alternatively due to interference by immunodominant peptides from other regions within p55^gag^, which are able to divert or inhibit immune responses targeting the conserved regions. Importantly, we showed that the use of p24CE as immunogen offers the advantage of focusing the immune responses to epitopes present within the highly Conserved Elements.

In this report, we expand our mouse immunogenicity study to examine the immunogenicity of p24CE DNA in macaques and demonstrate that the combination of p24CE DNA priming followed by p55^gag^ DNA boost provides a novel strategy to increase the magnitude and breadth of immune responses to Gag, including the induction of strong T-cell responses targeting epitopes within the highly Conserved Elements.

## Results

### Vaccination with p24CE DNA induces cellular immune responses to Conserved Elements in macaques

To focus the immune response to conserved targets of the HIV proteome, we tested a DNA vaccine expressing 7 highly conserved elements (CE) of 12–24 AA identified in HIV-1 p24^gag^ [32], [33] (**Figure 1A**) in macaques. The DNA vaccine (p24CE) expresses the collinearly arranged 7 CE (**Figure 1B**) by two vectors producing either p24CE1 or p24CE2 proteins, which differ by 1 ‘toggle’ AA per CE (**Figure 1A**). Together, these variant CE proteins cover >99% of the HIV-1 Group M sequence diversity and represent 54% of p24^gag^ sequence. To cover maximal responses, we only tested the immunogenicity induced by both immunogens in macaques. Ten animals were vaccinated using IM injection followed by *in vivo* electroporation (EP) with p24CE plasmids as outline in **Figure 1C** (left panel), using a mixture of p24CE1 and p24CE2 plasmids (N = 6) or a dual expression plasmid producing both CE immunogens (N = 4) (**Table 1**). The dual expression plasmid was generated to facilitate putative development of this DNA vaccine for clinical application. To measure the CE-specific responses, PBMC were stimulated with a mixture of 15-mer peptides (overlapping by 11 AA) and 10-mer peptides (overlapping by 9 AA) spanning the 7 CE to maximize the detection of CD4^+^ and CD8^+^ T cell responses. All 10 macaques developed CE-specific cellular responses, as measured by IFN-γ production, with a frequency ranging from 0.1% to 0.8% of total T cells in blood (**Figure 1C**). As expected, both the combination of the individual p24CE1 and p24CE2 DNAs or the dual expression plasmid producing p24CE1 and p24CE2 from a single molecule induced similar responses. Therefore, both single plasmids as well as the dual gene expression plasmid could be used for clinical development of this vaccine approach. These induced responses included both CD4^+^ and CD8^+^ T cells with 6 of the 10 vaccinated animals having dominant CD8^+^ T cell responses and 4 animals having primarily CD4^+^ T cell responses (**Figure 1C**).

**Table 1:** DNA vaccine regimens in rhesus macaques.

Macaque	DNA Delivery	Priming Vaccine (number of vaccinations)	Heterologous DNA Boost
L862	IM/EP	p24CE1+p24CE2 (2×)	COT-M p55^gag^
M166	IM/EP	p24CE1+p24CE2 (2×)	COT-M p55^gag^
M695	IM/EP	p24CE1+p24CE2 (2×)	COT-M p55^gag^
R279	IM/EP	p24CE1+p24CE2 (2×)	COT-M p55^gag^
R315^a^	IM/EP	p24CE1+p24CE2 (2×)	COT-M p55^gag^
P302^a^	IM/EP	p24CE1+p24CE2 (2×)	COT-M p55^gag^
P307^a^	IM/EP	p24CE1+p24CE2 (2×)	COT-M p55^gag^
P308^a^	IM/EP	p24CE1+p24CE2 (2×)	COT-M p55^gag^
P314^b^^,^ c	IM/EP	p24CE1+p24CE2 (3×)	COT-M p55^gag^
M437^b^^,^ c	IM/EP	p24CE1+p24CE2 (3×)	COT-M p55^gag^
L863	IM/EP	p24CE1+p24CE2+COT-M p55^gag^ (3×)	none
M629	IM/EP	p24CE1+p24CE2+COT-M p55^gag^ (3×)	none
P572	IM/EP	p24CE1+p24CE2+COT-M p55^gag^ (3×)	none
R285	IM/EP	p24CE1+p24CE2+COT-M p55^gag^ (3×)	none
L985	IM/EP	COT-M p55^gag^ (2×)	p24CE1+p24CE2
P574	IM/EP	COT-M p55^gag^ (2×)	p24CE1+p24CE2
R067	IM/EP	COT-M p55^gag^ (2×)	p24CE1+p24CE2
R288	IM/EP	COT-M p55^gag^ (2×)	p24CE1+p24CE2
M085^c^	IM/EP	HXB2 p37^gag^ (6×)	none
M114^c^	IM/EP	HXB2 p37^gag^ (6×)	none
M121^c^	IM/EP	HXB2 p37^gag^ (6×)	none
3169^c^	IM	HXB2 p55^gag^ (5×)	none
3274^c^	IM	HXB2 p55^gag^ (5×)	none
3278^c^	IM	HXB2 p55^gag^ (5×)	none
3290^c^	IM	HXB2 p55^gag^ (5×)	none

a Vaccine consists of a dual expression plasmid producing the p24CE1+p24CE2 antigens.

b Received prior 3 vaccinations with the DNAs expressing the poorly immunogenic unmodified p24CE.

c Vaccine did not include IL-12 DNA adjuvant.

### Vaccination with *gag* DNA induces poor cellular immune responses to Conserved Elements in macaques

We next investigated whether vaccination with a plasmid expressing native HIV-1 *gag* elicited CE-specific immune responses in 11 macaques, measuring the responses 2 weeks after the last *gag* DNA vaccination (**Table 1**). Upon peptide stimulation, all vaccinated animals showed anti-Gag cellular responses mediated by CD4^+^ and CD8^+^ T cells (**Table 2**), with overall levels comparable to those published by others using DNA IM delivery regimens [43]–[46]. The macaques mounted responses to the shared p24^gag^ epitopes (**Table 2**), revealing some animal-to-animal variation in the proportion of the p24^gag^ responses and elicited both p24^gag^-specific CD4^+^ and CD8^+^ T cells. Although all 11 macaques developed cellular immune responses that recognized p24^gag^–specific epitopes, only 5 of these macaques developed responses targeting CE (**Table 2**). Analysis of these responses revealed animal-to-animal differences with some skewing of the responses towards a primarily CD4^+^ or CD8^+^ T cell phenotype. Thus, these data, as noted in our mouse immunogenicity study [42], pointed to a difference between p24CE DNA and *gag* DNA vaccination in their ability to induce CE-specific responses.

**Table 2:** Cellular immune responses in HIV-1 *gag* DNA vaccinated macaques.

Animal	Gag DNA Vaccine	DNA Delivery	% Gag-specific T cells^a^		% p24^gag^-specific T cells^b^		% CE-specific T cells^c^	
			CD4^+^	CD8^+^	CD4^+^	CD8^+^	CD4^+^	CD8^+^
L985	p55^gag^	IM/EP	0.55	0.07	0.25	0.07	0.01	0.15
P574	p55^gag^	IM/EP	0.58	0.02	0.34	0	0.10	0
R067	p55^gag^	IM/EP	0.22	0.19	0.20	0.19	0.10	0.04
R288	p55^gag^	IM/EP	0.32	0.41	0.09	0	0.03	0
3169	p55^gag^	IM	0.12	0.21	0.07	0.01	0	0
3274	p55^gag^	IM	0.07	0.16	0.03	0.02	0	0
3278	p55^gag^	IM	0.02	0.09	0.01	0.09	0	0
3290	p55^gag^	IM	0.03	0	0.03	0	0	0
M085	p37^gag^	IM/EP	0.07	0.10	0.04	0.04	0	0
M114	p37^gag^	IM/EP	0.11	0.55	0.10	0.54	0	0
M121	p37^gag^	IM/EP	0.30	0.99	0.30	0.99	0.25	0

a analysis using peptide pool spanning p55^gag^ (15-mer overlapping by 11 AA).

b analysis using peptide pool spanning p24^gag^ (15-mer overlapping by 11 AA).

c analysis using peptide pool spanning all 7 CE (combination of 15-mer overlapping by 11 AA and 10-mer overlapping by 9 AA).

### p24CE DNA vaccination induces broader responses among CE

The responses to each of the CE were determined using peptide pools specific for the individual CE. Mapping the responses elicited by p24CE DNA vaccine revealed recognition of all CE except CE1 (**Table 3**). All 10 animals developed responses to CE5; seven macaques developed responses to 3 CE, two animals recognized 2 CE and one animal showed responses to only 1 CE.

**Table 3:** Higher response rate and broader responses among the 7 CE in the p24CE DNA vaccinated macaques.

Vaccine (Number of animals)		Cellular immune responses to individual CE^a^							Positive CE/animal		Response Rate
	ID	CE1	CE2	CE3	CE4	CE5	CE6	CE7	#	Range	
p24CE DNA (10)	L862		+	+		+			3	1–3	100%
	M166		+			+	+		3		
	M695			+		+	+		3		
	R279				+	+	+		3		
	P314			+	+	+			3		
	M437					+			1		
	R315					+		+	2		
	P302			+		+	+		3		
	P307			+		+	+		3		
	P308			+		+			2		
WT *gag* DNA (11)	L985			+		+			2	0–2	45%
	P574					+			1		
	R067					+	+		2		
	R288					+			1		
	M121					+	+		2		
	3169								0		
	3274								0		
	3278								0		
	3290								0		
	M085								0		
	M114								0		
p24CE+p55^gag^ (4)	L863		+			+	+		3	0–3	75%
	M629			+		+			2		
	P572								0		
	R285				+	+	+		3		

a CE responses were evaluated 2 weeks after the priming vaccination shown in Table 1 using a peptide pool spanning the 7 individual CE (combination of 15-mer overlapping by 11AA and 10-mer overlapping by 9AA).

Mapping of the CE responses in the subset of 5 *gag* DNA vaccinated macaques that elicited positive CE immunity (**Table 2**) showed that 2 animals recognized 1 CE, whereas 3 macaques developed responses to 2 CE, and all 5 animals shared responses to CE5 (**Table 3**). Of these animals, only animal L985 developed significant CE-specific CD8^+^ T cell responses, while the other 4 animals showed almost exclusively CD4^+^ T cell responses. The results from the *gag* DNA vaccination are in contrast to those obtained upon p24CE DNA vaccination, which elicited CE-specific responses in all 10 vaccinees mediated in the majority of the animals by CD8^+^ T cell responses (**Figure 1C**).

We also evaluated the association between CE responsiveness and MHC class I haplotype of the vaccinees. There was no apparent correlation between MHC class I haplotype in these out-bred animals and the ability to develop responses to CE (**Table S1**). Of note, the SIV Gag CM9 peptide sequence (CTPYDINQM) known to mount a dominant response in macaques with the MamuA01^+^ haplotype, is not conserved in HIV p24^gag^ CE vaccine (see CE2, **Figure 1A**) and there is no evidence of macaque haplotypes associated with the ability to mount a better response to HIV-1 Gag.

Importantly, the comparison of responses to epitopes within CE obtained upon p24CE and *gag* DNA vaccination in macaques showed that p24CE DNA vaccination induced responses in all vaccinees and showed responses to more CE/animal (median 3 CE/animal) (**Table 3**). In contrast, *gag* DNA vaccination showed a CE-specific responses rate of 45% with a range of 0–2 CE/animal. As suggested by our mouse study [42], the p24CE DNA vaccine induces broader immunity among the highly conserved epitopes of p24^gag^ also in macaques.

Two alternative hypotheses were formulated to explain the impaired CE recognition in most of the *gag* DNA vaccinated animals: (a) the immunogenicity of the CE epitopes within the Gag protein may be impaired due to suboptimal processing and presentation of the CE-containing peptides, or (b) immunodominance hierarchy favored variable regions within Gag and directed the CTL responses away from CE.

### p55^gag^ DNA vaccination boosts pre-existing CE-specific T cell responses

**Figure 2:**
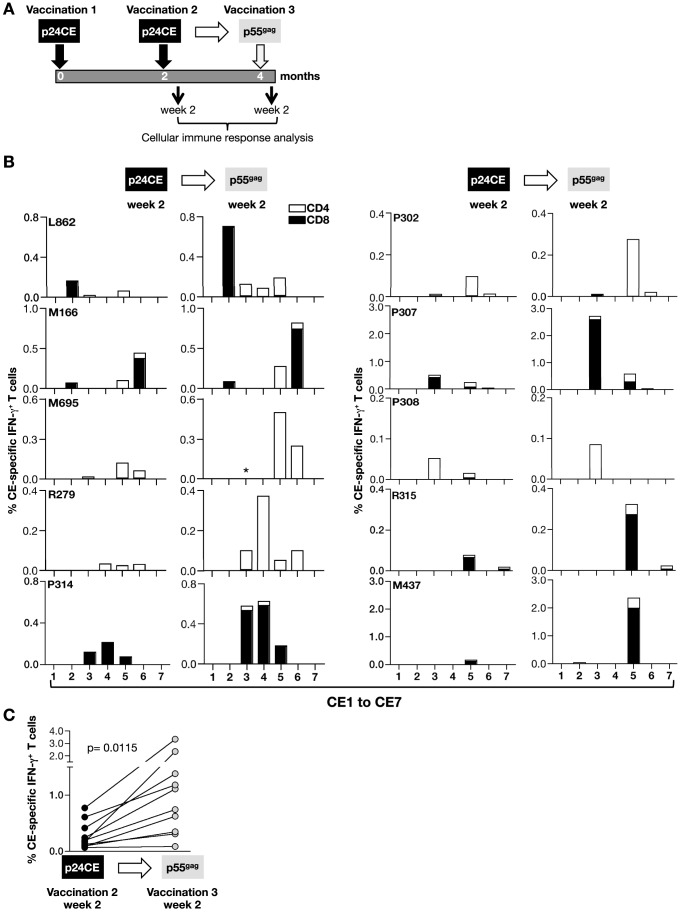
Boosting of p24CE DNA primed macaques with p55^gag^ DNA increases CE-specific cellular responses. (**A**) Vaccination schedule of the animals primed with p24CE DNA (Vaccination 1 and 2) and boosted with p55^gag^ DNA (vaccination 3). (**B**) Mapping of CE-specific T cell responses before (2 weeks after vaccination 2) and after (2 weeks after vaccination 3) the heterologous p55^gag^ DNA boost of the 10 vaccinated macaques. The percentage of IFN-γ^+^ CD4^+^ (open bars) and CD8^+^ (filled bars) T cells specific for each CE is shown. Note, although different scales were used for individual animals, the same scales are used before and after the DNA boost. Asterisks, indicates a sample that could not be analyzed. (**C**) Frequency of total CE-specific IFN-γ^+^ T cells before and after the heterologous boost. P values were determined using non-parametric Mann-Whitney test.

Given that repeated vaccination with p55^gag^ DNA failed or only poorly induced *de novo* CE-specific T cell responses (**Table 2** **and** **3**), we investigated whether a 3^rd^ vaccination using full-length *gag* DNA vaccination could boost and/or broaden pre-existing CE-specific immunity (**Figure 2A**; **Table 1**). The p24CE-vaccinated macaques received a vaccination boost with a plasmid expressing p55^gag^ DNA (**Figure 2A**). The responses were compared at 2 weeks after p24CE vaccination (vaccination 2, week 2) and 2 weeks after the *gag* DNA boost (vaccination 3, week 2). The complete set of pre-existing responses to individual CE was boosted in all 10 macaques (**Figure 2B**). In some animals (L862, R279), we noted the appearance of a new CE response. The *gag* DNA boost led also to a significant increase (p = 0.0115) in the frequency of IFN-γ^+^ CE-specific peak responses, reaching up to 2–3% of the total T cell population at 2 weeks after the boost (**Figure 2C**).

**Figure 3:**
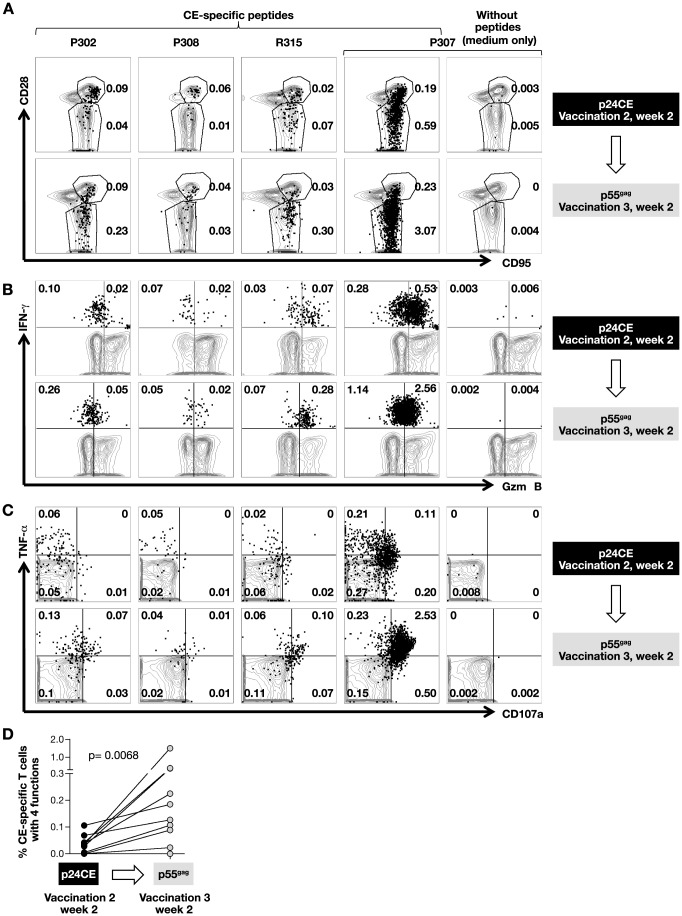
Characteristics of p24CE-induced T cells. Plot overlays show the phenotypic and functional characterization of the CE-specific T cells induced by p24CE DNA vaccination (2 weeks after vaccination 2; upper panels) and after the heterologous p55^gag^ boost (2 weeks after vaccination 3; lower panels) upon stimulation with CE-specific peptide pool. The analysis of macaque P307 is also shown in the absence of peptide stimulation (medium only) to illustrate the experimental background. The plots show overlays of total T cell population (grey contours) and the CE-specific IFN-γ^+^ T cells (black dots) with (**A**) central memory (CM; CD28^+^CD95^+^) and effector memory (EM; CD28^−^CD95^+^) phenotype; (**B**) granzyme B production and (**C**) TNFα and CD107a expression. The numbers in the plots represent the frequencies of CE-specific IFN-γ^+^ T cells. (**D**) Frequency of total CE-specific polyfunctional (4 functions; IFN-γ^+^ TNF-α^+^ CD107a^+^ GzmB^+^) CE-specific T cells before and after the heterologous boost. P values are from non-parametric Mann-Whitney test.

Analysis of CE-specific T cells showed that they were of both central (CD28^+^CD95^+^) and effector memory phenotype (EM, CD28^−^CD95^+^) with a predominant expansion of the effector memory subset upon p55^gag^ DNA boost (**Figure 3A**). Detailed analysis of CE-specific cellular immune responses showed that the p24CE DNA vaccine induced CD4^+^ and CD8^+^ T cells with cytotoxic potential targeting CE and that the elicited T cell responses were polyfunctional (4 functions) as defined by their granzyme B content, ability to secrete two cytokines (IFN-γ and TNF-α) and ability to degranulate (CD107a). Flow plots from 4 representative animals illustrate the increase of the granzyme B^+^ populations (**Figure 3B**) and of the TNF-α^+^ CD107a^+^ CE-specific T cells (**Figure 3C**). The frequency of CE-specific polyfunctional T cells was significantly augmented (p = 0.0068; **Figure 3D**), upon the heterologous p55^gag^ DNA boost (vaccination 3), reaching up to 1.5% of T cells. The CE-specific polyfunctional responses were not only limited to the CD8^+^ T cells (macaques R315, M437, P324) but were also found in several animals (M695, R279; R302, R308) which showed primarily CD4^+^ CE-specific T cell responses. Comparison of the responses before and after the DNA boost in these animals (M695: 0.038% to 0.225%; R279: 0.005% to 0.107%; R302: 0 to 0.023%; R308: 0.028% to 0.37%) showed that these responses greatly increased upon *gag* DNA boost (**Figure 2B**; **Figure 3D**). In agreement with our previous report on the induction of SIV-specific cytotoxic CD4^+^ T cells [47], [48], CE-specific cytotoxic CD4^+^ T cells could also be induced upon p24CE DNA vaccination and, importantly these responses could be augmented upon a single p55^gag^ DNA boost (vaccination 3) in macaques. Thus, this vaccine regimen was able to elicit features of a desired T cell vaccine.

Next, we examined whether pre-existing immunity to CE affected the development of *de novo* responses to other epitopes of Gag. Analysis of the responses to regions outside of p24^gag^ revealed that a single boost with p55^gag^ DNA induced responses to p17^gag^ and C-terminal regions of Gag (**Table 4**). Thus, pre-existing immunity to CE did not prevent development of *de novo* responses. Importantly, the successful boosting of the pre-existing CE responses by p55^gag^ DNA vaccination confirmed that the CE peptides were processed and displayed on the cell surface after vaccination with full-length Gag. Therefore, lack of immune response to CE in *gag* DNA vaccinated macaques was not related to absence of processing or presentation of CE-containing peptides, but rather to their inability to induce *de novo* responses in the presence of other, likely more dominant, Gag epitopes outside of CE. Together, our data showed that the hierarchy defined by Gag epitopes outside of CE was altered in the presence of pre-existing CE-specific responses. In conclusion, boosting of the p24CE DNA vaccinated macaques with *gag* DNA resulted in great increase in the responses to epitopes within individual CE and led to the induction of primary responses to less conserved epitopes as well.

**Table 4:** Single p55^gag^ DNA boost of p24CE DNA vaccinated macaques elicits *de novo* cellular immune responses to regions outside of the CE.

	Frequency (%) of IFN-γ^+^ T cell responses targeting different Gag regions:	
Macaque	p17^gag^	C' terminal Gag^a^
L862	0.02	0.01
M166	0.03	0
M695	0.03	0.20
R279	0	0.01
P314	0	0
M437	0	0
R315	0.03	0.01
P302	0.01	0
P307	0	0
P308	0	0.01

a analysis includes p2, p7, p1, p6 Gag peptides.

### Alternative vaccination regimens using p55^gag^ DNA prime followed by p24CE DNA boost or co-immunization with these two DNAs were less successful in inducing broad CE-specific responses

**Figure 4:**
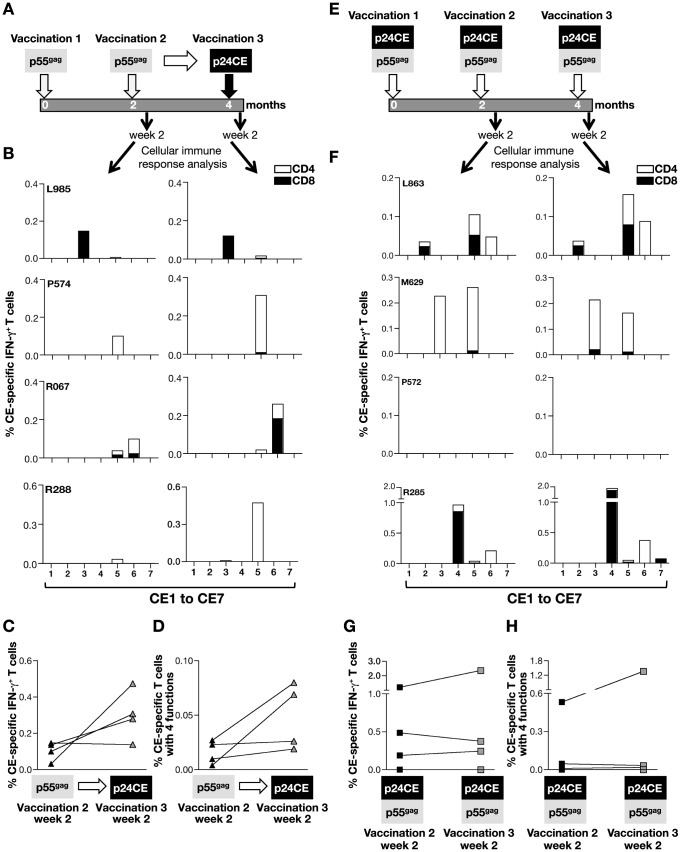
Changes of the CE response in macaques vaccinated with p55^gag^ DNA after heterologous boost with p24CE DNA and upon co-immunization with p24CE and p55^gag^ DNA. (**A**) Vaccination schedule of the animals primed with p55^gag^ DNA and boosted with p24CE DNA. (**B**) Four of the p55^gag^ DNA vaccinated animals, which developed cellular immune responses targeting CE (**Table 1**), received an additional vaccination with p24CE DNA. PBMC isolated 2 weeks after the 2^nd^ p55^gag^ DNA vaccination (vaccination 2) and after the p24CE DNA boost (vaccination 3) were stimulated with a CE-specific peptide pool (15- and 10-mer) covering individual 7 CE to map CE-specific T cell responses. The percentage of IFN-γ^+^ CD4^+^ (open bars) and CD8^+^ (filled bars) T cells specific for each CE is shown. (**C**) Frequencies of the total CE-specific IFN-γ^+^ T cells and (**D**) of the polyfunctional (4 functions; IFN-γ^+^ TNF-α^+^ CD107a^+^ GzmB^+^) CE-specific T cells are shown before and after the heterologous boost. (**E**) Vaccination schedule of the animals co-immunized with p24CE and p55^gag^ DNA. (**F**) PBMC isolated 2 weeks after the vaccinations 2 and 3 were stimulated with a CE-specific peptide pool covering the 7 individual CE. The percentage of IFN-γ^+^ CD4^+^ (open bars) and CD8^+^ (filled bars) T cells specific for each CE is shown. The frequencies of the total (**G**) CE-specific IFN-γ^+^ T cells and of the (**H**) polyfunctional (4 functions) CE-specific T cells are shown.

We further investigated 2 alternative vaccine regimens to explore the induction of CE-specific responses (**Figure 4A–D and 4E–H**). We compared the p24CE DNA prime-p55^gag^ DNA boost regimen (described in **Figure 2A**) to the reverse regimen of p55^gag^ DNA prime followed by p24CE DNA as boost (**Figure 4A**). We also tested the efficacy of p24CE and p55^gag^ DNA in a co-immunization regimen (**Figure 4E**).

First, we investigated whether p24CE DNA vaccination could alter the CE-specific immunity in macaques, which had developed CE responses after priming with p55^gag^ DNA. Four of the macaques vaccinated with p55^gag^ DNA (**Figure 4A** **;** **Table 2**), which showed CE-specific immunity were available. Comparison of peak CE-specific responses in the 4 macaques before (2 weeks after vaccination 2) and after the heterologous boost (2 weeks after vaccination 3) showed that the p24CE DNA boost increased the pre-existing CE-specific T cell responses (**Figure 4B–D**), although the difference did not reach statistical significance. The p24CE vaccination boosted the CE responses without altering the pre-existing CD4^+^ or CD8^+^ T cell distribution and also failed to elicit responses to new CE (**Figure 4B**). Compared to animals primed with p24CE, the p55^gag^ prime-p24CE DNA boost regimen maintained responses targeting one or two CE (**Figure 4B**). Thus, this vaccine strategy did not increase the breadth among the CE.

We further tested a co-immunization regimen using a mixture of p24CE and p55^gag^ DNA in four macaques (**Figure 4E**) and compared the responses 2 weeks after the 2^nd^ and 3^rd^ vaccination (**Figure 4F–H**). Only three of the four animals developed CE-specific responses with a range of 2–3 positive CE/animal (**Table 3**). We noted that maximal CE-specific responses were obtained upon 2 vaccinations (**Figure 4G**). Although this regimen provides the benefit of both vaccine modalities, the combination vaccine appears to dampen the individual effects. The co-immunization regimen did not induce CE-specific responses in all the animals (**Table 3**; **Figure 4F**) and did not elicit as broad responses within the Conserved Elements as obtained by the p24CE DNA prime (**Table 3**).

Together, comparison of the three vaccine regimens at 2 weeks after 3 vaccinations showed that the breadth of the CE-specific responses was distinct among the different vaccine regimens (**Table 3**). Our data demonstrate that a vaccine regimen using p24CE DNA prime followed by p55^gag^ DNA boost provides the maximal responses to epitopes within individual CE while also inducing responses to less well-conserved epitopes.

## Discussion

Several strategies have been tested to produce HIV vaccines addressing virus heterogeneity [1]–[17]. This report focuses on the analysis of a DNA vaccine expressing a set of highly conserved elements from HIV-1 p24^gag^. We demonstrate that vaccination of macaques with p24CE DNA vectors induces strong cellular immune responses targeting conserved epitopes within p24^gag^. We showed that p24CE DNA vaccination induces antigen-specific CD4^+^ and CD8^+^ T cells with cytotoxic potential and that the elicited T cell responses are mainly effectors with polyfunctional characteristics, which are desired features for an effective vaccine. Importantly, priming with p24CE DNA is critical to maximize CE responses (**Table 2**) and the inclusion of the *gag* DNA boost led to significant augmentation of the CE responses, while this vaccine regimen did not negatively impact on the overall p55^gag^ responses. Comparison of the three vaccine regimens after 3 vaccinations (2 weeks after vaccination 3) showed that all the groups developed similar robust total p55^gag^ responses, although the breadth of the CE-specific responses was distinct among different vaccine regimens (**Table 3**). Thus, vaccination using p24CE prime followed by *gag* DNA boost provides the maximal responses including improved breadth of the responses to epitopes within the individual CE.

We further demonstrate a clear advantage of the p24CE vaccine, showing maximal response rate as well as increased breadth and magnitude of responses within the conserved regions, which could not be achieved by vaccination with a *gag* DNA vector that express the conserved epitopes together with the variable regions. In contrast, Stephenson et al. [17] recently compared responses of full-length molecules to their conserved elements (Gag, Pol, and Env) vaccine and concluded that their conserved element vaccine did not provide any benefit (breadth or magnitude). The difference between the two studies may be due to the more strict definition and selection of our CE, which, in contrast to others, were selected in part by their association with virus control [33], further supporting their immunological relevance.

In this report, we experimentally tested the hypothesis that low immunogenicity of the conserved elements in the context of the natural protein sequence could be due to the presence of variable regions, which may exert an “immunodominant” decoy effect preventing the recognition of the conserved epitopes. In this context, the success of our p24CE prime-p55^gag^ boost study supports the concept that proper processing and surface display of CE-containing peptides from the native Gag protein takes place, and that these sets of CE containing peptides are recognized by T cells and are able to augment pre-existing CE responses. In addition, p55^gag^ boost induces *de novo* responses to regions outside the CE in macaques. These data imply that the hierarchy of epitope recognition established by p55^gag^ epitopes outside of CE was altered in the context of pre-existing CE-specific responses. This vaccine regimen suggests an approach to overcome a problem in the HIV vaccine field, where attempts to broaden the vaccine-induced immunity to include subdominant epitopes have been less successful using EP DNA/Ad boost immunization strategy [49] and a bias towards less-conserved regions was found in HIV-1 Ad5 gag/pol/nef vaccinated human volunteers [50] with responses in unique epitope hotspots which differed from those obtained from HIV infected individuals [51]. Together these reports point to the role of optimizing the vaccine design. In this context, our Conserved Element vaccine followed by complete immunogen boost may provide the long thought approach to overcome this obstacle.

Although full-length *gag* DNA vaccination induced only poor CE-specific responses in macaques, analyses of HIV-1 infected persons with different HLA haplotypes demonstrated the presence of CE-specific T cells during the chronic phase of infection [20], [33]. Higher avidity CTL responses in these regions were identified in HIV controllers and detailed analysis of the responses demonstrated that, for most epitopes analyzed, controllers were able to recognize more peptide variants [33]. This indicates that TCR promiscuity could be beneficial for the recognition of epitopes with mismatched amino acids resulting in better control of viral replication and prevention of escape mutants. These data also suggest that high avidity CE-specific responses are a potential correlate of HIV control. It is not clear why vaccination with full-length Gag generates poor CE responses [mice [42] and macaques (this report)], while these responses are detected in chronic HIV infection. It would be of interest to study different vaccination regimens and also to examine the time of development of CE responses during natural infection. The difference in elicited immune response is reminiscent of a previous report by Ferrari et al. [52], who showed that the immunodominant p17^gag^ SL9 response identified in HLA-A*0201 infected persons could not be induced upon ALVAC-gag vaccination in these haplotype-selected volunteers, although this epitope has been implicated in the sieve effect observed in the STEP HIV vaccine trial [53]. Both studies suggest that there may be differences between vaccine-induced and infection-induced cellular responses that should be taken into consideration for successful vaccine design; they also highlight the potential immunodominant decoy effect of a full-length immunogen design.

The question then arises whether a T cell vaccine could benefit from the responses elicited by conserved T cell epitopes. Our CE DNA vaccine was selected to focus immunity to highly conserved sequences in a haplotype-independent manner [33]. A recent report by Mudd et al. [54] demonstrated that Mamu-B*08 macaques vaccinated with a T cell vaccine eliciting CD8^+^ T-cell responses targeting 3 different viral epitopes were able to efficiently control SIV_mac239_ replication even in the absence of Env responses. In one of the animals, loss of control of viremia correlated with the emergence of escape mutant virus. The CE vaccine may provide advantages by focusing responses that are not easily overcome by virus escape in an outbred population.

The presented results contribute to the development of improved vaccine strategies against HIV, targeting the immune responses to highly conserved viral regions. We hypothesize that cellular immune responses targeting conserved regions of HIV or other highly variable pathogens (e.g. influenza, hepatitis C, dengue, yellow fever), which do not allow rapid escape mutations without significant loss of viral fitness, thereby inducing immunity targeting the ‘Achilles heel’ of the virus, are more likely to be protective. A recent report by Valkenburg et al. [55] described preemptive priming, an approach aiming to prevent the development of known escape mutants, taking into consideration the direction of immune responses to highly restricted essential regions and thereby focusing the responses to critical epitopes of the virus. Since there is evidence that vaccine-induced responses can change upon HIV infection resulting in virus escape in humans [56], a selection of conserved elements avoiding epitopes that may act as immunodominant decoys is of importance for the design of an effective vaccine. Importantly, we demonstrate a novel concept that combination of conserved elements and full-length immunogen alters the immunological hierarchy and allows for the development and expansion of subdominant responses.

A successful vaccine should be able to generate potent cross-clade specific humoral and cellular responses against conserved regions of the virus. Our work provides an effective strategy to overcome restrictions associated with immunodominance, while improving the magnitude and breadth of responses, especially those against conserved regions, minimizing the possibility of viral escape while increasing the recognition of naturally occurring divergent HIV strains. Our results indicate that a vaccine candidate should be designed to extend this concept to the entire HIV proteome. Since the macaque model was found in general to provide a similar response hierarchy to that obtained upon vaccination of humans, our macaque study supports the further evaluation of the novel CE vaccine strategies in man.

## Materials and Methods

### DNA vectors

The 7 CE selected within the p24^gag^ region were connected via short linker sequences designed for efficient proteolytic cleavage and were linked to the human GM-CSF signal peptide at the N-terminus as described [42]. Plasmids pSP-p24CE1 (plasmid 234H) and pSP-p24CE2 (plasmid 235H) contain the expression-optimized p24CE open reading frames inserted in vector pCMVkan [57], comprising a plasmid backbone optimized for growth in bacteria, the human cytomegalovirus (CMV) promoter without any introns, the optimized p24CE genes, the bovine growth hormone (BGH) polyadenylation site, and the kanamycin resistance gene. Plasmid p24CE1+p24CE2 (plasmid 306H) expresses both of the p24CE genes from a dual promoter plasmid [58], where SP-p24CE1 is expressed from the human CMV promoter and SP-p24CE2 from the simian CMV promoter arranged in counter clock-wise orientation. The COT-M p55^gag^ [59] DNA (plasmid 222H) expresses a full-length Gag representing a center-of-tree sequence from an RNA/codon optimized gene. HXB2 *gag* is expressed from optimized genes as p55^gag^ or p37^gag^ (p17gag+p24gag). The IL-12 DNA (plasmid AG157) produces the rhesus macaque IL-12 cytokine from an optimized expression vector [58]. Endotoxin-free DNAs (Qiagen, Valencia, CA) were prepared according to the manufacturer's protocol.

### Vaccination of macaques

Animals were vaccinated with a mixture of 1 mg of each of SP-p24CE1 and SP-p24CE2 (L862, M166, M695, R279), 2 mg of the dual expression plasmid p24CE1+p24CE2 (P302, P307, P308, R315) or 1 mg of COT-M p55^gag^ DNA (L985, P574, R067, R288) together with 200 µg of macaque IL-12 DNA formulated in 0.6 ml of sterile water (Hospira, Inc., Lake Forest, IL). DNAs were delivered via intramuscular (IM) injection at two different sites (0.3 ml each site) followed by *in vivo* electroporation using the Elgen 1000 device (Inovio, Pharmaceuticals, Inc, Blue Bell, PA). The animals received two vaccinations with p24CE DNA or p55^gag^ DNA (EP1, EP2 at 0 and 2 month) followed by converse vaccination with p55^gag^ DNA or p24CE DNA (EP3 at 4 month), respectively. Four animals (L863, M629, P572, R285) received 3 vaccinations with a combination of 1 mg each of SP-p24CE1 and SP-p24CE2 DNAs plus 1 mg COT-M p55^gag^ DNA together with 200 µg IL-12 DNA. Blood samples were collected at the day of each vaccination and 2 weeks later. Two additional macaques (M437 and P314) were included in the p24CE group, after receiving sequentially the poorly immunogenic unmodified p24CE DNAs [42] followed by 3 SP-p24CE DNA vaccinations. Immune response analysis from 7 additional macaques [60], [61] vaccinated with HXB2 *gag* DNA was included in this study: 4 animals (3169, 3274, 3278 and 3290) had received p55^gag^ DNA by IM injection [61], and 3 animals (M085, M114 and M121) had received p37^gag^ DNA (p17^gag^+p24^gag^) by IM injection followed by *in vivo* electroporation [60], as reported previously. Blood samples collected 2 weeks after the last vaccination were analyzed.

### Intracellular cytokine staining

Macaque PBMC were isolated by Ficoll-hypaque (Histopaque, Sigma, St. Louis, MO) centrifugation, and cultured in 96-well plates as described previously [62]. PBMC were incubated in the presence of various peptide pools and the frequency of antigen-specific T cells was measured by intracellular cytokine staining followed by polychromatic flow cytometry. Antigen-specific cellular immune responses were measured using the following peptide pools: CE-specific peptide pools covering all or the 7 individual CE were composed of a mixture of 15-mer Group M peptides overlapping by 11 AA (#11057, AIDS Research and Reference Reagent Program, Germantown, MD) and 10-mer peptides overlapping by 9 AA (Peptide Synthesis Facility, Massachusetts General Hospital, Boston) covering both p24CE1 and p24CE2 proteins; Group M consensus 15-mer Gag peptides overlapping by 11 AA, spanning the 3 regions p17^gag^, p24^gag^ and the C-terminal p2, p7, p1, p6 Gag proteins, respectively at a final concentration of 1 µg/ml for each peptide. After an overnight incubation with monensin (BD Pharmingen, San Diego, CA), cells were stained with the following cocktail of cell surface antibodies: CD3-APCCy7 (clone SP34-2), CD4-V500 (clone L200), CD95-FITC (clone DX2) (BD Pharmingen), CD8-Alexa Fluor-405 (clone MHCD0826, Invitrogen, Carlsbad, CA), CD28-PerCP Cy5.5 (clone CD28.2, BioLegend, San Diego, CA), CD45RA-AF700 (clone F8-11-13, ABD Serotec, UK). In some experiments, CD107a-Alexa Fluor 647 (clone H4A3, eBioscience San Diego, CA) antibody was added to the cells 10 min after addition of the peptides. After permeabilization of the cells using the Cytofix/Cytoperm kit (BD Biosciences), intracellular staining was performed using IFN-γ-PE Cy7 (clone B27, BD Pharmingen) and Granzyme B-PE antibodies (clone GB12, Invitrogen). The 4-function analysis included TNF-α-Alexa Fluor 700 (clone Mab11, BD Pharmingen). In all experiments, PBMC stimulated with phorbol myristate acetate (PMA) and calcium ionophore (Sigma, St. Louis, MO) were used as positive control. A negative control sample, consisting of PBMC cultured in medium without peptide stimulation, was included for each macaque analyzed. Samples were considered positive, if the frequency of IFN-γ^+^ T cells in the peptide stimulated sample was at least 2 fold higher than the frequency obtained in unstimulated (minus peptide) medium only control sample (see **Figure 4A–C**) and the responses must be higher than 0.01% after subtracting the values obtained from the negative control. At least 10^5^ T cells from each sample were acquired on an LSR II flow cytometer (BD Biosciences, San Jose, CA) and the data were analyzed using FlowJo software (Tree Star, Inc., Ashland, OR).

### Statistical analysis

Analyses were performed using Prism 6 (GraphPad Software, Inc, La Jolla, CA) and the P values were calculated using the non-parametric two-tailed t-test (Mann-Whitney).

### Ethics statement

This study was carried out in accordance with the Guide for the Care and Use of Laboratory Animals of the National Institutes of Health. Rhesus macaques were housed and handled in accordance with the standards of the Association for the Assessment and Accreditation of Laboratory Animal Care International at the Advanced BioScience Laboratories Inc., and were approved by the Institutional Animal Care and Use Committee (OLAW assurance number A3467-01 and USDA Certificate number 51-R-0059).

## Supporting Information

Table S1 **Haplotype of vaccinated macaques and recognition of CE.**
(DOCX)
