# Correction: Altered Response Hierarchy and Increased T-Cell Breadth upon HIV-1 Conserved Element DNA Vaccination in Macaques

**DOI:** 10.1371/journal.pone.0103198

**Published:** 2014-07-14

**Authors:** The PLOS ONE Staff

The complete ethics statement for this article was not published. The publisher apologizes for this error. A complete version of the ethics statement is provided here:

The animals in this study were Indian rhesus macaques (Macaca mulatta) and were housed at the Advanced BioScience Laboratories, Inc. (ABL) animal facility. All animals were cared for and procedures performed under a protocol approved by the ABL Animal Care and Use Committee (animal welfare assurance no. A3467-01; protocol no. AUP516). Furthermore, the macaques in this study were managed according to the animal husbandry program of the ABL Animal Facility, which aims at providing consistent and excellent care to nonhuman primates at the vivarium. This program operates based on the laws, regulations, and guidelines promulgated by the United States Department of Agriculture (e.g., the Animal Welfare Act and its regulations, and the Animal Care Policy Manual), Institute for Laboratory Animal Research (e.g., Guide for the Care and Use of Laboratory Animals, 8th edition), Public Health Service, National Research Council, Centers for Disease Control, and the Association for Assessment and Accreditation of Laboratory Animal Care (AAALAC) International. The nutritional plan utilized by the ABL Animal Facility consisted of twice daily feeding of Labdiet 5045 High Protein Primate Diet and food intake was closely monitored by Animal Research Technicians. This diet was also supplemented with a variety of fruits, vegetables, and other edible objects as part of the environmental enrichment program established by the Veterinary staff and enrichment Technician. Pairing of animals as part of the environmental enrichment program was managed by the enrichment technician. All primary enclosures and animal rooms were cleaned daily with water and sanitized at least once every two weeks. All macaques used in this study were males, except for four females: M695, P572, P574, M437. Their average weight was 8.5 kg (range: 3.8-11.2 kg) and their average age was 7 years (range: 4-12 years). Vaccinations were performed under anesthesia (Ketamine administered at 10 mg/kg) and all efforts were made to minimize suffering. None of the animals were euthanized as part of this study.
