# Supplementary material for: Altered Response Hierarchy and Increased T-Cell Breadth upon HIV-1 Conserved Element DNA Vaccination in Macaques
Source: PLoS One. 2014 Jan 23;9(1):e86254. doi: 10.1371/journal.pone.0086254 (PMC3900501; doi:10.1371/journal.pone.0086254)
Supplement: Table S1 — Haplotype of vaccinated macaques and recognition of CE. (DOCX) [file pone.0086254.s001.docx]

**Table S1.** Haplotype of vaccinated macaques and recognition of CE

| Vaccine | **Animal** | **A*01** | **A*02** | **A*08** | **A*11** | **B*01** | **B*03** | **B*04** | **B*08** | **B*17** | **CE Recognized^#^** | | | | | |
| --- | --- | --- | --- | --- | --- | --- | --- | --- | --- | --- | --- | --- | --- | --- | --- | --- |
| p24CE DNA | L862 | Neg | Neg | Neg | Neg | Neg | Pos | Neg | Pos | Neg | CE2 | CE3 |  | CE5 |  |  |
|  | M166 | Neg | Pos | Neg | Neg | Pos | Neg | Neg | Neg | Neg | CE2 |  |  | CE5 | CE6 |  |
|  | M695 | Neg | Pos | Neg | Neg | Pos | Neg | Neg | Neg | Pos |  | CE3 |  | CE5 | CE6 |  |
|  | R279 | Pos | Neg | Neg | Neg | Neg | Neg | Neg | Neg | Pos |  |  | CE4 | CE5 | CE6 |  |
|  | P314 | Neg | Neg | Neg | Neg | Neg | Neg | Pos | Neg | Pos |  | CE3 | CE4 | CE5 |  |  |
|  | M437 | ND | ND | ND | ND | ND | ND | ND | ND | ND |  |  |  | CE5 |  |  |
|  | R315 | Pos | Neg | Neg | Neg | Neg | Neg | Neg | Neg | Neg |  |  |  | CE5 |  | CE7 |
|  | P302 | Neg | Neg | Neg | Neg | Pos | Neg | Neg | Neg | Pos |  | CE3 |  | CE5 | CE6 |  |
|  | P307 | Neg | Neg | Neg | Neg | Neg | Neg | Pos | Neg | Neg |  | CE3 |  | CE5 | CE6 |  |
|  | P308 | Neg | Neg | Neg | Pos | Pos | Pos | Neg | Pos | Neg |  | CE3 |  | CE5 |  |  |
| p55^gag^ DNA^$^ | L985 | Neg | Neg | Neg | Neg | Neg | Neg | Pos | Neg | Neg |  | CE3 |  | CE5 |  |  |
|  | P574 | Neg | Pos | Neg | Neg | Neg | Neg | Neg | Neg | Neg |  |  |  | CE5 |  |  |
|  | R067 | Pos | Neg | Neg | Neg | Neg | Neg | Neg | Neg | Pos |  |  |  | CE5 | CE6 |  |
|  | R288 | Pos | Neg | Neg | Neg | Pos | Neg | Neg | Neg | Neg |  |  |  | CE5 |  |  |
|  | M121 | Pos | Neg | Neg | Pos | Neg | Neg | Neg | ND | Neg |  |  |  | CE5 | CE6 |  |
| p24CE DNA+  p55^gag^ DNA co-immunization | L863 | Neg | Neg | Pos | Neg | Neg | Neg | Neg | Neg | Neg | CE2 |  |  | CE5 | CE6 |  |
|  | M629 | Neg | Pos | Neg | Neg | Pos | Neg | Neg | Neg | Neg |  | CE3 |  | CE5 |  |  |
|  | P572 | Neg | Pos | Neg | Neg | Neg | Neg | Neg | Neg | Neg |  |  |  |  |  |  |
|  | R285 | Pos | Neg | Neg | Neg | Neg | Neg | Neg | Neg | Neg |  |  | CE4 | CE5 | CE6 |  |

ND, not done

^$^includes only 5 of the 11 p55^gag^ DNA vaccinated macaques which showed CE responses.

**^#^**data from Table 3.
